# Spinal manipulative therapy versus Graston Technique in the treatment of non-specific thoracic spine pain: Design of a randomised controlled trial

**DOI:** 10.1186/1746-1340-16-12

**Published:** 2008-10-30

**Authors:** Amy Crothers, Bruce Walker, Simon D French

**Affiliations:** 1 School of Chiropractic and Sports Science, Murdoch University, 90 South Street, Murdoch, Western Australia, Australia; 2 Monash Institute of Health Services Research, Monash University, Melbourne, Australia

## Abstract

**Background:**

The one year prevalence of thoracic back pain has been estimated as 17% compared to 64% for neck pain and 67% for low back pain. At present only one randomised controlled trial has been performed assessing the efficacy of spinal manipulative therapy (SMT) for thoracic spine pain. In addition no high quality trials have been performed to test the efficacy and effectiveness of Graston Technique^® ^(GT), a soft tissue massage therapy using hand-held stainless steel instruments. The objective of this trial is to determine the efficacy of SMT and GT compared to a placebo for the treatment of non specific thoracic spine pain.

**Methods:**

Eighty four eligible people with non specific thoracic pain mid back pain of six weeks or more will be randomised to one of three groups, either SMT, GT, or a placebo (de-tuned ultrasound). Each group will receive up to 10 supervised treatment sessions at the Murdoch University Chiropractic student clinic over a 4-week period. Treatment outcomes will be measured at baseline, one week after their first treatment, upon completion of the 4-week intervention period and at three, six and twelve months post randomisation. Outcome measures will include the Oswestry Back Pain Disability Index and the Visual Analogue Scale (VAS). Intention to treat analysis will be utilised in the statistical analysis of any group treatment effects.

**Trial Registration:**

This trial was registered with the Australia and New Zealand Clinical Trials Registry on the 7^th ^February 2008. Trial number: ACTRN12608000070336

## Background

Published studies of the epidemiology of non-specific thoracic pain are uncommon. Niemelainen found that the one year prevalence of mid back pain in Finnish men was 17%, compared to 64% with neck pain and 66.8% who reported low back pain [1]. When upper or mid back pain was present, disability tended to occur less than if the pain was reported in the neck or low back. However, when disability was reported, the number of days of disability was similar when the pain involved the upper or mid back compared to other regions.

Commonly used treatment options for non specific thoracic spine pain include massage, mobilisation, manipulation, acupuncture, and other physical therapies such as heat, electro-therapies, ultrasound and also non steroidal anti-inflammatories. A search of the literature concerning thoracic spinal pain established that there are no high quality studies for any of these modalities. There are some individual studies, however none show unequivocal proof of efficacy or effectiveness.

To date we are only aware of one published randomised controlled trial performed assessing the effectiveness of spinal manipulative therapy on thoracic spinal pain [1]. Spinal manipulative therapy (SMT) was compared to a placebo group receiving non functional ultrasound in a small trial consisting of 30 patients with "mechanical" thoracic spine pain. The authors reported mixed results. The SMT group demonstrated significantly better reductions in numerical pain ratings and improvements in lateral flexion at the end of a two to three week treatment period. These findings were maintained at one month follow up, however at this point they were no longer statistically significant. Concurrently there were no significant differences between groups in McGill Pain Questionnaires and Oswestry Pain Disability Indices at any point of the trial. Limitations such as a small sample size and inadequate follow up were acknowledged by the author. This, in combination with a lack of trials undertaken in this area, provides further evidence that future trials are warranted in determining the efficacy of SMT for the treatment of non specific thoracic pain.

For the purposes of this study we have chosen to review the following modalities; spinal manipulative therapy and Graston technique^® ^(GT). The reasons for these choices are that spinal manipulation is a very common treatment worldwide and GT is a popular massage technique in the United States and becoming more popular in other developed countries. In addition, GT is taught and used routinely at the Murdoch University Chiropractic Clinic.

Soft tissue or massage therapy is described by Walker et al as a very popular method for the treatment of low back pain in a general population [2]. At present, research has been undertaken to assess the effects of massage therapy on pain, function and patient satisfaction in adults with mechanical neck and low back pain [3]. However, no evidence exists for this same modality in the treatment of non specific thoracic pain.

Graston Technique^® ^is a massage system revolving around several hand-held stainless steel instruments. The promoters of the Graston Technique [4] claim that the instruments are much like tuning forks as they reportedly resonate in the clinician's hands allowing the clinician to isolate adhesions and restrictions, and treat them very precisely. The promoters also claim that the metal surface of the instruments do not compress the tissues, as do the fat pads of the finger, so that deeper restrictions can be accessed and treated. However, we are not aware of any high level evidence to support any of these claims. There are six numbered instruments of different shapes and sizes designed for different areas of the body.

At present, limited research has been undertaken to determine the effectiveness of GT. Only one pilot study exists in which two manual therapy techniques were utilised: Graston instrumented soft tissue mobilisation and soft tissue mobilisation administered by the clinician's hands in the treatment of carpal tunnel syndrome [5]. While no differences were observed between the two manual therapies, both showed improvements in nerve conduction latencies, wrist strength and wrist motion. These improvements were maintained at 3 months follow up. There was no comparison with a placebo or sham treatment.

In terms of spinal musculature, one case study has been published in the treatment of sub-acute lumbar compartment syndrome with Graston Technique [6]. Following 6 treatments, the patient experienced a full resolution of the complaint and fascial extensibility was restored.

Given the overall lack of scientific evidence it is apparent that further high quality trials are necessary to determine the efficacy of the soft tissue massage method known as Graston Technique and SMT. We propose a study to determine the efficacy of SMT compared to Graston Technique in the treatment of non specific thoracic pain.

## Methods and design

This study will be a randomised, placebo-controlled trial comparing two different treatment modalities to an intervention of no known benefit for people with acute or sub-acute thoracic spine pain. The therapy arms will consist of SMT and Graston Technique (GT) and the placebo will be non-functional ultrasound. A placebo group will be utilised because at present there are no proven treatments for non specific thoracic pain. Reporting of this trial will adhere to the CONSORT statement [7-9] and is registered with the Australia and New Zealand Clinical Trials Registry [10]. Ethics approval has been granted by Murdoch University Human Research and Ethics Committee, number 2007/274.

The aim of this three arm trial is to test the efficacy of SMT and GT as independent modalities compared to detuned ultrasound for the outcomes of pain and disability measured using the Visual analogue scale (VAS) and a modified Oswestry Back Pain Disability Index.

### Study sample and participant enrolment

**Figure 1:**
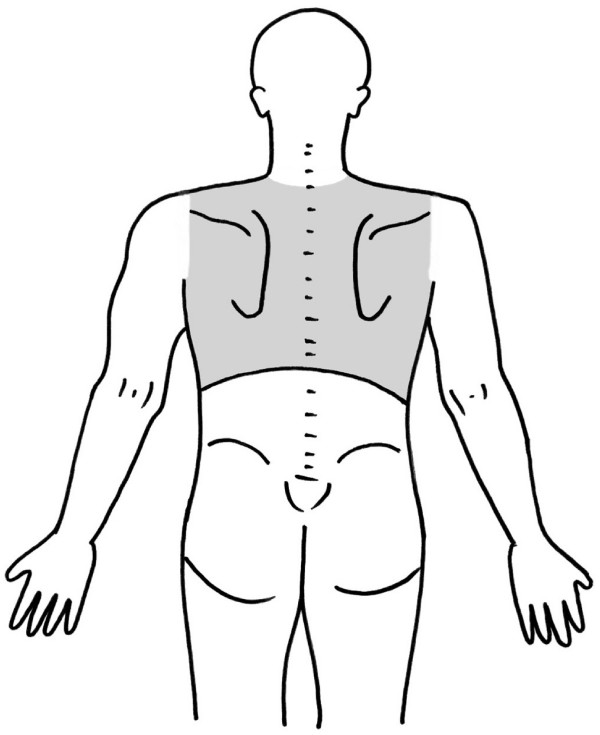
Definition of thoracic spine pain area.

The study will be conducted at the Murdoch University Chiropractic student clinic in Perth, Australia. Participants will be recruited by the use of advertisements posted around the Murdoch University Campus, on local community boards and in newspapers. Any person who responds to the advertisement with symptoms consistent with non-specific thoracic spine pain and meets the inclusion criteria in the screening checklist via phone interview will be considered a potential participant for the study. Participants will be at least 18 years old with a primary complaint of thoracic pain (Figure 1: Mannequin defining the area of thoracic pain) and with no contraindications to manual therapy or Graston Technique.

**Figure 2:**
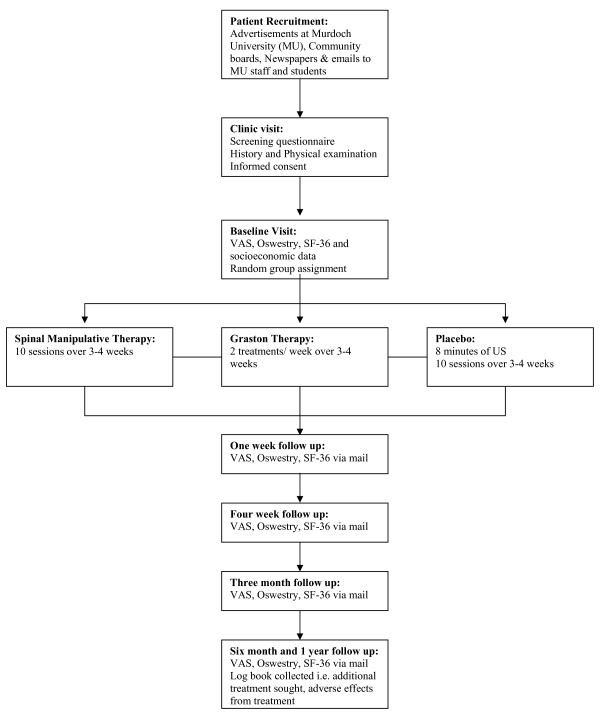
Flow chart of study.

Upon making an appointment at the Murdoch University Chiropractic Clinic subjects will be screened and investigated with a detailed history and physical examination by Research Assistant A (a trained final year chiropractic student). The potential patients will be considered suitable candidates for the study if they meet all the inclusion criteria and none of the exclusion criteria are found. At this point, participants who agree to enter the trial will read and sign an informed consent form that will be administered and witnessed by Research Assistant A. All persons who enter the trial will be properly accounted for and attributed at the conclusion of the trial. Refer to Figure 2 for a flow diagram of the methods.

### Inclusion Criteria

People will be included into the trial if they meet the following criteria:

1. Age 18 years or older with non-specific thoracic spine pain, which is pain in the region from T1 to T12 (Figure 1) and complies with the descriptive classification by Triano et al [11] (Table 1). For the purpose of this trial our definition will include an area outside the midline.

**Table 1:** Definition of non specific Thoracic spine pain [11]

**Definition of non specific Thoracic spine pain**
Midline back pain – for the purposes of this trial, the pain will be bound by the lateral margins of the thorax laterally and the trapezium superiorly
Non dermatomal referred pain difficult to localise
No signs of nerve root tension
No major neurological deficit
Pain with compression over the thoracic spine into spine extension
Reduced range of motion

2. A VAS score of 2 out of 10 [12] or greater and an Oswestry Back Pain Disability index score of greater than 15% at baseline [13].

### Exclusion Criteria

People will be excluded if they meet any of the following exclusion criteria:

1. Have a contraindication to manual therapy (inclusive of osteoporosis, thoracic fracture, spinal infection, neoplastic disorders, spondyloarthropathy, clinical examination suggestive of frank disc herniation or generalised infection such as influenza).

2. Have contraindications to Graston technique (inclusive of neoplastic disorders, kidney infection, anticoagulant medication, rheumatoid arthritis, uncontrolled hypertension, thoracic fracture, osteomyelitis or generalised infection)

3. Have somatic conditions found on examination to refer pain to the thoracic spine from outside the defined area (inclusive of cervical zygapophyseal joints, muscles and discs)

4. Have an active history of visceral conditions referring pain to the thoracic spine (inclusive of myocardial ischaemia, dissecting thoracic aortic aneurysm, peptic ulcer, acute cholecystitis, pancreatitis, renal colic, acute pyelonephritis)

5. Have a current substance abuse problem.

6. Are not fluent and/or literate in the English language

7. Are currently receiving care for thoracic pain from any other provider

8. Cannot commit to the full study protocol

9. Are currently seeking compensation or have commenced litigation for thoracic spine pain

Radiographs will be taken when deemed necessary to exclude patients with contraindications to manipulation or other complicating disease, as described in the exclusion criteria. The decision to use x-ray will be based on the criteria set by the National Health and Medical Research Council for acute thoracic pain [14].

### Treatment allocation

Randomisation will occur directly after baseline measures are undertaken and after the subject has been screened for inclusion and the history, physical examination, report of findings and consent has been completed. An online randomisation site, Research Randomiser [15], will be used to generate treatment allocation. This online randomisation module is a web browser application that supports online randomisation of patients into healthcare trials. Research Randomizer uses the "Math.random" method within the JavaScript programming language to generate its random numbers. To prevent delay in the processing of participants, 100 random sequences will be generated, and then the sequences will be placed in sequentially numbered, opaque, sealed envelopes and stored in a sealed box. As each participant enters the trial the next consecutive opaque, sealed envelope will be given to the treating student and supervising clinician by Research Assistant A. This will allocate the treatment the participant is due to receive. Allocation is kept secure and concealed in the envelope until the patient information has been entered and the person requesting the randomisation has confirmed that they wish to proceed with entry into the trial. The participants will be analysed in the groups to which they are randomised using intention to treat analysis.

### Interventions and treatment

Participants will be randomised to one of three treatment arms as follows:

1. Chiropractic group: a series of chiropractic manual adjustments (SMT) to the thoracic spine administered by a registered chiropractor or a final year chiropractic student under the direct supervision of a registered chiropractor.

2. Graston Technique group: Graston Technique will be administered by final year chiropractic students who have been certified in module one of the Graston Technique, under the direct supervision of a registered chiropractor who will attend each consultation and place their hands on the anatomical regions involved;

3. Placebo group: participants will receive a session of de-tuned ultrasound administered by a final year chiropractic student, under the direct supervision of a registered chiropractor who will attend each consultation and place their hands on the anatomical regions involved.

All treating students will be trained to show the same enthusiasm for all three treatment modalities. The time taken for each consultation for the three treatment modalities will be approximately the same and last about 10 – 15 minutes. In the group that receives SMT, the spinal level(s) selected for SMT will be at the discretion of the student using usual chiropractic diagnostic methods including both motion and static palpation (application of pressure to tolerance directly over the facet joints) [16]. The SMT itself consists of taking the joint slack to the elastic barrier and a high-velocity low-amplitude thrust delivered at the level, and in the direction, of the notional loss of joint motion (fixation). Low velocity techniques and mobilisation will not be considered as manipulation for the purposes of this protocol. The SMT will be considered to be successful if the registered chiropractor who is supervising the final year student is satisfied with the administration and delivery of the manipulation itself. The participants will receive a maximum of 10 sessions of manipulation over a minimum period of 3 weeks to a maximum period of 4 weeks with 3 to 4 sessions per week. This dosage of treatment is based on the randomised controlled trial undertaken by Haas et al [17] who concluded that relief in low back pain intensity and functional disability was most substantial in those patients who received chiropractic treatment 3 to 4 times per week for 3 weeks.

The participants allocated to the Graston Technique Protocol will receive treatment as required to thoracic spine musculature inclusive of the Rhomboids Major and Minor, Upper and Lower Trapezius, Latissimus Dorsi and Levator Scapulae. The Graston Technique treatment will be administered by students who have successfully completed a Level 1 Graston training program and will involve the use of the patented form of instrumented-assisted soft tissue massage/mobilisation. The treating student will initially scan the area for notional "muscle adhesions" using the Graston instruments. Once the tissue area of interest has been localised instruments three and six will be utilised to apply deeper pressure for approximately 1 to 2 minutes to the area of concern. The participants will be scheduled to receive 10 treatments over a period of 3 to 4 weeks complying with that recommended by the developers of the technique [4].

The placebo group will be given eight minutes of detuned ultrasound for up to 10 sessions over a 3 to 4 week period. This control modality will be used as there is no known treatment benefit from the detuned machine; however, it has been established in previous trials that participants view this as a credible treatment option [18,19]. To increase the perceived credibility, the treating student will place one hand on area adjacent to the participants' involved mid-back region while delivering the treatment. The participants will be treated with the same enthusiasm as other treatment groups. Participants in this group will also undergo an examination including routine screening for contraindications at the first consultation and the normal clinical reassessment that would occur at subsequent treatments as per the intervention groups. Each placebo treatment session will be about 10–15 minutes in duration to match the active treatment sessions. Following the review period of 12 months, participants in this group will be offered the treatment of their choice (SMT or GT).

Participants will be monitored to ascertain whether their pain levels have increased beyond their baseline measurement or reduced to no pain at all. In such cases, the participants who cease to receive treatment due to an increase in pain will be noted and followed up. This occurrence will also be noted under adverse effects. On the other hand those who recover completely will be noted as a success. All participants will be followed up over the 12 months.

### Outcome measures and baseline data

Socio-demographic and other possible prognostic factors will be collected at baseline. Socio-demographic variables include age, sex, race/ethnicity, education, household income, marital status, and current employment status. General health status will be measured at baseline with the 36-item Short-Form Health Survey (SF-36) which has 8 scales: physical function, role physical, bodily pain, general health, vitality, and social function, role emotional and mental health. These can be aggregated into two summary measures: physical and mental health. Questions regarding previous episodes of thoracic pain and number of episodes in the last 2 years will be asked in the initial screening questionnaire.

Two main self assessment outcome measures will be used in this study; a 100 mm VAS for the participant's perception of pain intensity and a modified Oswestry Back Pain Disability Index to measure the participant's perceived disability and function.

The VAS is a tested and established instrument for the assessment of pain [20,21]. It consists of a horizontal line 10 cm long with the words "no pain" at one end and "pain as bad as it could be" at the other. Participants are asked to indicate which point along the line best represents their pain intensity. The distance from the no-pain end to the mark made by the participant is their pain intensity score. The Oswestry Back Pain Disability Index (ODI) has also been validated for the low back [22,23] and consists of 10 items assessing the level of pain interference with physical activities (e.g. pain intensity, lifting and travelling) resulting in a disability percentage. If some single items are not answered, the overall score is computed from the available information. For the purpose of this study, the Oswestry Back Pain Disability Index will be modified slightly, changing the words 'leg pain' to read 'back pain as seen in the diagram provided'. We acknowledge that with this change we cannot make the assumption that the instrument is reliable and valid for pain and disability in the thoracic spine. However it is likely on the face of it that the instrument is transferable from low back to mid back application.

Main outcome measures (VAS and ODI) will be taken at baseline, one week after treatment commences, upon completion of the 4-week intervention period and at three, six and 12 months post randomisation. Outcome assessments completed on the day of randomisation will be given to an independent research assistant B (not involved in treatment delivery) and placed in a secure box to be assessed only by the research co-ordinator. A statistician blinded to group allocation will analyse the data. At the first consultation a package will be given to the participants with the remaining five assessments to be completed on the dates advised in the package. A text message will be sent to the participant on the day that the outcome is due to be completed as a reminder to fill it out and return it back to the student clinic via a stamped envelope contained in the package. If the outcomes are not returned in a timely manner, an additional copy of the outcome measures will be sent to participants encouraging them to complete the forms.

### Adverse Effects

Participants will be provided with a list of potential adverse effects in an information letter prior to giving to consent. Information about adverse events and side effects will be collected in the form of a log book which will be handed to the participants in a package following their initial consultation. This will allow the participant to note any adverse effects, how long it lasted, a pain rating out of 10 and how often it occurred. Participants responses to the question of adverse effects will be collected at the six month follow up stage. At six and twelve months we will also collect data from the log books on additional treatment received for their mid back pain after the treatment intervention period.

### Blinding

The primary outcome measures are self-administered instruments that will be distributed by a research assistant following the initial consultation. The participants of the study will be given blank questionnaires in a package following their first treatment to be completed at each assessment point. After completion of the forms the participant will seal them in an envelope provided in the package and post them back to the Murdoch University Chiropractic Clinic. The envelopes will then be placed in a secure box for the research co-ordinator to retrieve. Research assistants will remain blind to the outcome data for the entire study period and will be continually counselled by the research investigator regarding the importance of blinding. They will be trained in administration of informed consent and outcome data retrieval. To facilitate quality control throughout the study, regular meetings with relevant questions by the research co-ordinator of the assistants will help to prevent incidents of unblinding. The participants and treatment providers will not be blinded to the treatment allocation as it will be clear that the groups are receiving different treatments Participants in the placebo group will be blinded to their placebo allocation until follow-up is complete at 12 months. Participants will be surveyed for the adequacy of the placebo blinding at the six month review.

### Data analysis

The patient's data will be coded to ensure that no one involved with data analysis will be aware of the treatment provided.

Descriptive statistics will be used to check the data. Outliers and inconsistencies will be followed up by reviewing paper records and contact with study participants.

Summary statistics of demographic and potential confounding variables will be calculated and presented by intervention group at baseline. Multiple linear regression models will be used to compare the interventions for the primary outcomes, pain and disability, at one and four weeks and three, six and twelve months. For the respective models, the baseline of these outcomes will be included as an explanatory variable. Adjustment for baseline of the outcome using regression analysis is generally the preferred approach because of beneficial statistical properties [24]. In addition, adjustment will be made for pre-specified potential confounders. These will be included in models even when no baseline imbalance exists. Additional exploratory analyses will be undertaken using random effects models to compare the effect of the interventions over time. No adjustment will be made for multiple testing.

A detailed statistical analysis plan will be written prior to completion of the trial including details of the models to be implemented and the multiple imputation strategy for handling missing data [25].

### Sample size calculations

To calculate the sample size, we used the means of 23.9, 18.9 and 13.9 and assumed a standard deviation of 12.1 of the ODI (primary outcome measure) derived in a study by Hoiriis et al [24] in a similar chiropractic teaching setting. The clinical effect size used for the ODI was 10% [13], alpha was set at .05 and power at .80. Sample size was calculated at n = 30 for each of the three groups. This was calculated using a commercial package, nQuery Advisor [26]. The clinically significant difference for the VAS ranges between 10 and 14 mm [12]. Sample size calculations using the VAS resulted in a smaller n for each group, therefore the ODI was used.

## Discussion and conclusion

This paper outlines the rationale and design for a randomised controlled trial that compares the effectiveness of SMT and Graston Technique to the placebo treatment of non-functional ultrasound in the treatment of non-specific thoracic pain. The primary outcomes to be measured will be the participants perceived pain intensity using the Visual Analogue Scale and also the participants perceived disability level by means of the Oswestry back pain disability index. This trial aims to provide further evidence for the treatment of non-specific thoracic pain.

## Abbreviations

SMT: Spinal Manipulative Therapy; GT: Graston Therapy; US: Ultrasound.

## Competing interests

The authors declare that they have no competing interests. BW is Editor in Chief of Chiropractic & Osteopathy and SF is an associate editor. Neither was involved in the peer review process for this manuscript.

## Authors' contributions

AC, BW and SF were responsible for the conception and the design of the study. All authors read and approved the final manuscript. AC is running this trial as part of her Honours Degree in chiropractic.
